# Efficacy of the porcine species in biomedical research

**DOI:** 10.3389/fgene.2015.00293

**Published:** 2015-09-16

**Authors:** Karina Gutierrez, Naomi Dicks, Werner G. Glanzner, Luis B. Agellon, Vilceu Bordignon

**Affiliations:** ^1^Department of Animal Science, McGill University, Sainte-Anne-de-BellevueQC, Canada; ^2^School of Dietetics and Human Nutrition, McGill University, Sainte-Anne-de-BellevueQC, Canada

**Keywords:** Large animal models, biomedical research, swine, pigs, minipigs, clones, transgenics

## Abstract

Since domestication, pigs have been used extensively in agriculture and kept as companion animals. More recently they have been used in biomedical research, given they share many physiological and anatomical similarities with humans. Recent technological advances in assisted reproduction, somatic cell cloning, stem cell culture, genome editing, and transgenesis now enable the creation of unique porcine models of human diseases. Here, we highlight the potential applications and advantages of using pigs, particularly minipigs, as indispensable large animal models in fundamental and clinical research, including the development of therapeutics for inherited and chronic disorders, and cancers.

## Introduction

**FIGURE 1:**
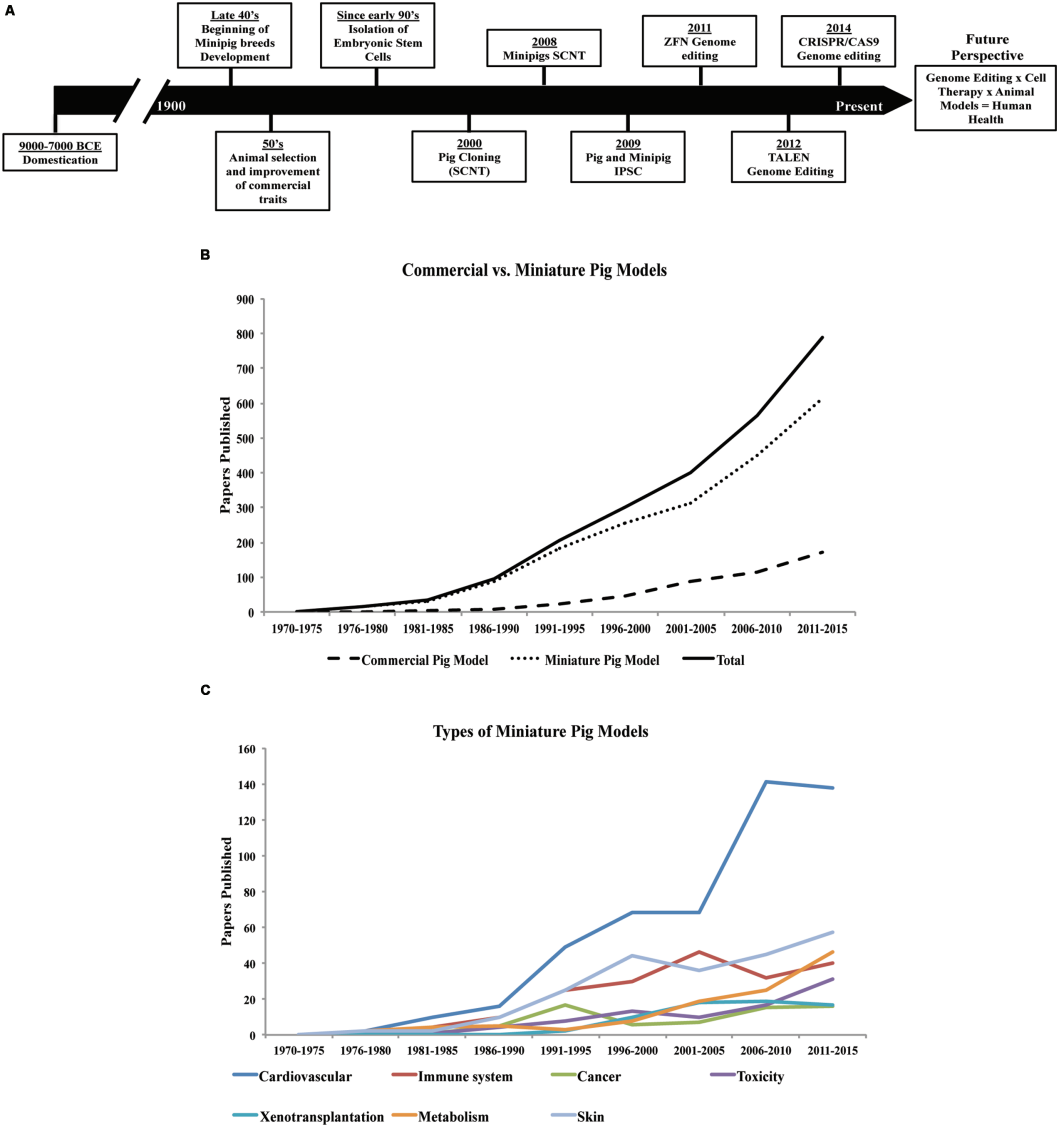
**History of pigs in agriculture and research since domestication. (A)** Timeline, significant events and application of different technologies in the selection and breeding of the porcine species (Jones, 1998; Onishi et al., 2000; Polejaeva et al., 2000; McGlone and Pond, 2003; Brevini et al., 2007a,b; Estrada et al., 2008; Safranski, 2008; Wakai et al., 2008; Esteban et al., 2009; Ezashi et al., 2009; Hauschild et al., 2011; Köhn, 2011; Larson et al., 2011; Carlson et al., 2012; Hai et al., 2014; Whitworth et al., 2014). **(B)** Use of the porcine species in research, and **(C)** application of minipig models in a variety of studies (based on articles indexed by PubMed, from 1970 to the present date).

The first evidence of swine domestication dates back to approximately 7000–9000 years ago (Jones, 1998; McGlone and Pond, 2003; Köhn, 2011; Larson et al., 2011; **Figure 1A**). China and Europe have been, since domestication, the pig-breeding centers dictating the profile of the pig breeds (Jones, 1998; Amills et al., 2001). The reason for domestication was to provide meat as a source of food protein, which stimulated pig selection and farming (Jones, 1998; Köhn, 2011). Studies have been conducted using genome-wide genotyping and genetic variability to trace the migration, selection, and improvement from ancient wild species to modern swine (Giuffra et al., 2000; Bosse et al., 2014a,b). It is generally accepted that the majority of all modern breeds are derived from the Eurasian wild boar (European and Asian wild boars; Porter, 1993; Bosse et al., 2014b). Although pig selection started just after domestication, it has only been since the mid-20th century that performance has been used as the main tool in the animal selection process (Safranski, 2008). More recently, molecular biology technologies, genome-wide association studies, and next-generation sequencing have been applied to enhance the selection process of domesticated pig breeds (e.g., Duroc, Landrace, Pietrain, Yorkshire, etc.) to further improve traits of high economic value such as feed conversion, meat quality, growth, precocious puberty, and prolificity (Sahana et al., 2013; Tart et al., 2013; Jiang et al., 2014; Sanchez et al., 2014).

The variety of modern pig breeds available today (Buchanan and Stalder, 2011), are a product of human intervention since domestication, but especially during the last century (**Figure 1A**). Besides breeds specialized for food production, smaller sized breeds (miniature- and micro-pigs) with certain characteristics such as obedience, friendly nature, and cognitive ability have also been selected for the purpose of companion animals. In addition, their use in biomedical research has been increasing considerably in the last years (**Figure 1B**).

Compared with other animals used in research (e.g., mice, rats, rabbits, and dogs), domestic farm pigs are much larger (>300 kg adult size), therefore, requiring more space and feed, and making them harder to handle. Mini- or micro-pigs are hence more desirable for research use. The adult sizes vary among breeds, reaching around 20–30 kg for a Panepinto micropig to 100 kg for a Munich minipig (Köhn, 2011). Although many minipig breeds are a product of crossbreeding, some breeds, like the Yucatan pigs, are naturally occurring stocks (Panepinto, 1996; Köhn, 2011). Since the late 1940s, minipigs have been further developed specifically for biomedical research purposes (England and Panepinto, 1986; Köhn, 2011).

There are now several minipig breeds available for use in research (Panepinto, 1996). The main breeds developed in the USA are Yucatan, Sinclair (also known as Minnesota or Hormel miniature pig), Hanford, NIH minipig and Panepinto miniature pig. The minipig breeds developed in Europe are Göttingen, Munich, Berlin, Mini-Lewe, Czech-Republic, Vietnamese potbellied and Mini-Sib. In Asia, the breeds include Ohmini, Clawn, Lee Sung, and Chinese minipigs. The Göttingen and Yucatan breeds are the most commonly used minipigs in research, although there is no apparent clear reason for preference. Unlike the Yucatan, a natural breed, the Göttingen minipig was developed specifically for research use. Other breeds are used only by specific research groups, thus limiting their widespread availability in research. Nevertheless, the interest in the use of pigs in biomedical research has been rising over the last 40–45 years (**Figure 1B**).

## Use of Pigs in Biomedical Research

Biomedical research is broad, spanning studies on underlying disease mechanisms to the evaluation of safety and effectiveness of preventative measures, diagnostic tests, and therapies. Most animal studies in recent times have used the murine species due to their small size, fast reproductive cycles and short lifespan. In addition, the availability of murine embryonic stem cells, fully annotated genome, and facile tools for targeted genetic manipulation have all contributed to the elucidation of gene functions and disease pathophysiology. However, in many cases, mouse models do not adequately represent features of human disorders (Seok et al., 2013). In this regard, animals that better represent human pathophysiology are required. Pigs and humans share many similarities such as size, physiology, anatomy, metabolic profile, and longer lifespan (Panepinto, 1996; Spurlock and Gabler, 2008; Kuzmuk and Schook, 2011; Swindle et al., 2012). For example, pig skin is structurally similar to human skin regarding thickness and spacing between hair follicles, making it useful for studies on wound healing and burn lesions (Sullivan et al., 2001). Pigs also share anatomical and physiological similarities with respect to the renal system, making them valuable for pharmacological studies (Dalgaard, 2014; Huppertz et al., 2015). Pigs can also be useful in the study of nutrient absorption and intestinal transport, as well as the pathogenesis of gastrointestinal diseases (Sangild et al., 2014). All these characteristics contribute to the development of superior models of human conditions (Kuzmuk and Schook, 2011).

The choice between outbred or inbred strains can have a significant impact on research outcomes (Festing, 2014). While, outbred strains may be better suited for quantitative trait loci studies, experiments addressing mechanistic aspects would benefit from the use of inbred strains (Chia et al., 2005). Some minipig breeds are already established for specific applications due to their unique characteristics (**Table 1**). Pigs have also been used for testing new therapies, devices, and efficacy and safety of new drugs prior to human trials. For instance, a novel endovascular chemotherapy filter, designed to reduce circulatory drug excess *in vitro*, was successfully tested in pigs (Patel et al., 2014). As well, a new method for pediatric liver transplantation was validated using pigs (Leal et al., 2015). Regarding pharmacokinetic and cytotoxic tests, pigs have been used for testing topical skin formulations (Mitra et al., 2015), and are considered a better choice compared to dogs for the study of drugs that are metabolized by the aldehyde oxidase (AOX1), *N*-acetyltransferase (NAT1 or NAT2) or cytochrome (CYP2C9-like) enzymes (Dalgaard, 2014).

**Table 1:** Characteristics and applications of minipig breeds for the study of human conditions.

Parameter	Yucatan	Gottingen	Hanford	Sinclair/Minnesota
Adult body size (kg)	70–83	~45	80–95	55–70
Average litter size	6	6.5	6.7	7.2
Age to puberty (months)	4–6	3–5	4–6	4–6
Genetic background	Purebred	Outbred	Outbred	Outbred
Cloning	somatic cell nuclear transfer (SCNT; Estrada et al., 2008)	SCNT (Wakai et al., 2008)	Information not available	SCNT (Do et al., 2012)
Transgenics	Homologous recombination *BRCA1* (breast cancer susceptibility gene 1) – gene knockout by rAAV – model for breast cancer (Luo et al., 2011, 2012)^∗^ Introduction of missense mutation via rAAV – *TP53* gene – cancer cells (Sieren et al., 2014) Introduction of nonsense mutation via rAAV – *SCN5A* gene – cardiac arrhythmia (Park et al., 2015)	Homologous recombination *BRCA1* (breast cancer susceptibility gene 1) – gene knockout by rAAV – model for breast cancer (Luo et al., 2012)^†^ rAAV vectors encoding GFP (Kornum et al., 2010)	Information not available	ZFN – mono and biallelic knockout pigs – *CMAH* gene – xenoantigen involved in the rejection phenomenon (Kwon et al., 2013) TALEN – biallelic modified pigs – *RAG2* gene – immune system (Lee et al., 2014)
Applications	Wound healing (Eggleston et al., 2000) Cardiovascular model for ventricular septal defect (VSD; Swindle et al., 1990) Metabolic Disorder (Phillips et al., 1982)	Toxicity Studies (Bollen and Ellegaard, 1997; van Mierlo et al., 2013) Skin pharmacokinetics tests (Mitra et al., 2015) Metabolic Syndrome (Johansen et al., 2001) Neurodegenerative disease – Parkinson Model (Bjarkam et al., 2008) Obesity (Christoffersen et al., 2013) Heart failure (Schuleri et al., 2008)	Dermal studies – toxicology (Leigh et al., 2012) Wound healing (Reger et al., 1999) Surgery training (Purohit et al., 1993) Tests of new therapies in tissue regeneration (Van Dyke et al., 2015)	Oncology (malignant spontaneously regression melanoma; Oxenhandler et al., 1979) Dermatology – skin depigmentation (Millikan et al., 1974) Models of human alcoholism (Dexter et al., 1976) Pediatric hypothyroidism (Tank et al., 2013)

*^∗^The animals died 18 days after birth.*

*^†^Cloned animals were not yet born at the time of publication.*

In general, there is low incidence of naturally occurring pathologies described in pigs. The reason for this is twofold. First, human intervention by way of selective breeding has eliminated genes that increased disease susceptibility. Second, the majority of the domestic farm pigs are slaughtered at a young age (< 6 months old), precluding the detection of late onset diseases such as cancer. On the other hand, Vietnamese potbellied minipigs raised as companion animals do reach old ages. Indeed, a retrospective study found a variety of neoplasms with widespread metastases in these pigs of advanced age (~11 years; Newman and Rohrbach, 2012). The most common malignances found included hepatic and intestinal carcinomas, and uterine and ovarian smooth muscle tumors (Newman and Rohrbach, 2012).

Occurrence of malignant spontaneously regressing melanomas has been described in Sinclair minipigs (Millikan et al., 1974; Oxenhandler et al., 1979). Selective interbreeding, by removing animals with red coat color that do not develop the lesions, increased the frequency of tumor formation in these selected minipigs (Millikan et al., 1974). The tumors appear from birth and culminate in skin depigmentation after tumor regression showing a phenotype similar to human vitiligo (Millikan et al., 1974). Studies conducted in these minipigs have shown decreased telomerase activity during melanoma regression (Pathak et al., 2000), which has also been observed by inhibiting telomerase activity in human melanoma cells (Burchett et al., 2014). Therefore, these minipigs may represent a useful model to study malignant melanomas because the tumors appear spontaneously and then either regress or grow progressively and metastasize similarly to human melanomas (Oxenhandler et al., 1979).

Another example of a naturally occurring condition in pigs is the dwarf phenotype, caused by a single amino acid change in the α1 chain of type X collagen (Nielsen et al., 2000). The *COL10A1* gene, which encodes type X collagen, is expressed in hypertrophic chondrocytes during endochondral ossification. In humans, an amino acid variation in the same position of the type X collagen protein has been shown to be the cause of Schmid metaphyseal chondrodysplasia (SMCD), a mild skeletal disorder associated with dwarfism (Warman et al., 1993). Since mice lacking type X collagen do not develop abnormalities in long bone development (Rosati et al., 1994), pigs represent a better animal model of human SMCD.

Another naturally occurring disease observed in Yucatan minipigs mimics human ventricular septal defect (VSD; Swindle et al., 1990). The VSD in pigs can be observed in fetal stages similar to the congenital anomaly in humans, and can be used for the study of new methods of diagnosis or therapies (Swindle et al., 1990; Amin et al., 2006).

Despite a number of natural occurring pig phenotypes that resemble human diseases, for most of human pathologies it is difficult to find representative animal models in nature. Thus, manipulation of diet, use of drugs and/or surgeries has been necessary to generate appropriate models. For example, minipig models for Type I diabetes were induced via administration of streptozotocin or alloxan to selectively destroy insulin-producing cells (Phillips et al., 1980; Larsen et al., 2002). High-energy diets in young minipigs lead to the development of obesity and metabolic syndromes, with increased visceral fat deposition, glucose intolerance, decreased insulin sensitivity, and higher levels of blood cholesterol and triglycerides, which progress to Type 2 Diabetes mellitus (Xi et al., 2004; Neeb et al., 2010; Koopmans and Schuurman, 2015). Other chemicals have been used to induce cellular dysregulation and damage in pigs including the administration of *N*-nitrosodiethylamine to produce a liver cancer model (Li et al., 2006).

## Use of Engineered Pigs in Biomedical Research

Genetically modified animals have been instrumental in advancing our understanding of gene function and significance of inappropriate gene expression in metabolic malfunction in mammals. Genome editing holds great promise in generating these models, and has already permitted the rapid development of new pig models of several human diseases (Rogers et al., 2008; Prather et al., 2013; Hai et al., 2014; Dicks, 2015).

The cystic fibrosis (CF) model is an example of genetically engineered pigs created by targeted inactivation of the cystic fibrosis transmembrane conductance regulator (*CFTR*) gene (Rogers et al., 2008). The resulting pigs exhibit clinical features and disease progression consistent with those observed in CF infants. In contrast, inactivation of the *CFTR* gene in mice did not produce the comorbidities typically observed in human CF patients (Snouwaert et al., 1992).

Advanced reproductive technologies, such as somatic cell nuclear transfer (SCNT), can now be routinely applied to large animal species, including minipigs. Minipigs of different breeds have been cloned from different cell types, including genetically modified cells (Estrada et al., 2008; Kurome et al., 2008; Wakai et al., 2008; Zhao et al., 2009). In addition SCNT offers the possibility of creating isogenic and immunocompatible animals from the same cell line. Importantly, models of severe disorders can be generated from engineered cultured cells without the need of breeding sick animals. The sequencing of the pig genome is another key development in the production of gene-modified pigs in the post-genomic era (Schook et al., 2015a). Genome editing techniques, including zinc finger nucleases (ZFN), transcription activator-like effector nucleases (TALEN), and clustered, regularly interspaced, short palindromic repeats (CRISPR) together with CRISPR associated (Cas) nucleases (CRISPR/Cas), now allow the precise manipulation of gene sequences in germ, embryonic and somatic cells (Hauschild et al., 2011; Carlson et al., 2012; Cong et al., 2013; Hai et al., 2014; Whitworth et al., 2014; Dicks, 2015). Among these methods, the CRISPR/Cas9 system is emerging as the method of choice because it permits gene editing to be accomplished in only one step by injecting both the specific guide RNAs and endonuclease into zygotes (Hai et al., 2014; Whitworth et al., 2014).

Another example of human disease that has the potential to be studied in genetically engineered pigs is heart arrhythmias (Park et al., 2015). Mutations in the *SCN5A* gene, which encodes a subunit of the cardiac sodium channel Na_v_1.5 required for excitability and conduction in the myocardium, were found in patients with Bruguda syndrome (Hedley et al., 2009). *SCN5A^E558X/+^* engineered Yucatan minipigs with reduced expression of the sodium channel Na_v_1.5 have been created and these animals exhibit conduction abnormalities and susceptibility to ventricular arrhythmias (Park et al., 2015). There has also been considerable interest in genetically modified pig strains suitable for xenotransplantation. Most research into the development of appropriate xenotransplantation strains focused on addressing hyperacute rejection, which is initiated rapidly and involves preformed natural human antibodies and the complement system (Cooper et al., 2002). This has been possible by targeting cell surface antigens such as α-1,3 galactosyltransferase (Miyagawa et al., 2001; Lai et al., 2002; Phelps et al., 2003; Takahagi et al., 2005) or complement regulatory proteins such as human decay accelerating factor (Murakami et al., 2002). The pigs made deficient of α-1,3 galactosyltransferase have contributed to the reduction of immunogenicity of donor tissue/organs (Phelps et al., 2003). Transgenic pigs expressing antibodies against cytotoxic T-cell lymphocyte antigen receptor, a cell-mediated immune response suppressor, were also developed (Phelps et al., 2009).

A pig model for the human familial adenomatous polyposis was generated by inactivation of the adenomatous polyposis coli (*APC*) gene (Flisikowska et al., 2012). Mice lacking the *APC* gene exhibit non-metastatic neoplasias only in the small intestine (Su et al., 1992). However, the pig model of colon and rectal cancer reproduces the human features of the disease, which includes the development of polyps spread along the whole large bowel in young animals. A candidate gene for the development of breast and ovarian cancer models is the breast cancer-associated gene 1 (*BRCA1*), which has been manipulated in both Yucatan and Göttingen cells, but lines of modified minipigs remain to be produced (Luo et al., 2011, 2012). The *TP53* gene, which encodes the tumor suppressor protein p53 and is the most commonly observed suppressed gene in human tumors, was found to be mutated in Li-Fraumeni patients having increased risk to develop multiple types of cancers (Gonzalez et al., 2009). Suppression of p53 in mesenchymal stem cells derived from pig bone marrow exhibits chemoresistance *in vitro* (Leuchs et al., 2012). Mutation of *TP53* gene in Yucatan minipigs resulted in development of lymphomas and osteogenic tumors (Sieren et al., 2014). More recently, a new engineered pig strain termed “oncopig” was developed, which promises inducible formation of a wide variety of cancers that are potentially novel platforms for research and therapeutics development (Schook et al., 2015b). These examples illustrate the potential of genetically engineered pigs as robust models for the study of human pathologies that are not well represented in small laboratory animal species.

## Improving the Usefulness of Pigs in Biomedical Research

Rodents have been the choice animal model for basic research, but are not always suitable for translational research due to marked differences in size, lifespan as well as metabolic, anatomical, and physiological discrepancies. On the other hand, the pig is more closely related to humans in terms of these parameters (Swindle et al., 2012) and, therefore, is better suited for recapitulation of human diseases. Indeed, the use of the pig in translational research is increasingly gaining acceptance (**Figure 1C**). Dogs and non-human primates have traditionally been used for this purpose, but rising ethical concerns have reduced their favor and increased demand for alternatives (Swindle et al., 2012). The number of peer-reviewed papers describing the use of pigs as biomedical models has risen eightfold over the past 30 years (**Figure 1B**). Already, the pig has become well established in many areas of research and training. For instance, in the past 20 years the pig has replaced the dog as a model for surgical training and has also gained FDA approval for the testing of surgical implantation devices intended for human use (Swindle et al., 2012; Schook et al., 2015a). Minipig models, which are much smaller in size compared to the domestic farm breeds, offer lower operating costs compared to other large animal models and also reduce the concern of ethical acceptance given the already widespread use of pigs in agriculture (Bollen and Ellegaard, 1997; Swindle et al., 2012).

Pigs offer many exciting applications, including stem cell research, tissue engineering and xenotransplantation. Although incredible advances in transgenic pigs harboring various engineered alterations designed to minimize graft versus host rejection (Lai et al., 2002; Phelps et al., 2003, 2009; Klose et al., 2005; Takahagi et al., 2005; Hauschild et al., 2011; Petersen et al., 2011; Jeong et al., 2013), much work remains to be accomplished since multiple genes need to be manipulated given the various types of tissue rejection reactions (Takahagi et al., 2005; Whyte and Prather, 2011; Jeong et al., 2013). Porcine induced pluripotent stem cells (iPSCs) have been produced (Esteban et al., 2009) and chimeric pigs were generated using iPSC (West et al., 2010, 2011). This is highly relevant since study of porcine iPSCs have eventual human applications (Esteban et al., 2009), such as cell-based therapies. However, the mechanisms of cellular reprogramming, directed cell differentiation and species-specific cell culture requirements necessitate further investigation (Ezashi et al., 2012). The International Society for Stem Cell Research has indicated in their guidelines for translational use that validation must occur in both small and large animal models (Aigner et al., 2010). Tissue repair is another potential application of engineered pig models. Cartilage tissue grafts have been created using chondrocytes isolated from infant minipigs (Deponti et al., 2014), and mandibular condyle grafts have been generated from Yucatan minipig adipose-derived mesenchymal stem cells (Abukawa et al., 2003). There has also been successful regeneration of bone defects using engineered bone graft tissues in minipig models (Gröger et al., 2003). If custom donor transgenic minipig strains can be created, this could open the doors to other engineered tissue replacements for human uses. For example, the use of blastocyst complementation and pluripotent stem cells has been applied to direct the development of otherwise missing organs in pigs (Matsunari et al., 2013). This has increased the hope that it may one day be possible to create non-immunogenic donor organs in pigs using human iPSCs (Matsunari et al., 2013; Feng et al., 2015). Finally, similarities in the porcine and human immune system have sparked interest in vaccine development and efficacy testing in pigs (Meurens et al., 2012).

The completion of the porcine genome project in 2012 has further facilitated the use of pigs in research. Data from this project has enabled the comparative analysis of genetic sequences and development of the necessary tools to create and validate targeted genetic alterations in the porcine genome (Gun and Kues, 2014; Schook et al., 2015a). In addition, the development of RNASeq technology has facilitated transcriptome analysis, which further improves our ability to identify important targets related to certain phenotypic traits (Ropka-Molik et al., 2014). Other recent achievements in the pig include the use of inducible or conditional systems to control transgene expression (Kues et al., 2006; Klymiuk et al., 2012), and tissue-specific expression of the Cre recombinase (Li et al., 2009; Luo et al., 2014). These advances will ensure the continued development of various pig strains for research, similar to what has already been accomplished in mice.

## Summary

It is clear that the use of the pig as a biomedical model is increasingly gaining approval due to physiopathological similarities with humans. However, some obstacles remain to be overcome in order to realize the full potential of the porcine species in developing new diagnostic and therapeutic approaches. Despite the sequencing of the porcine genome, full annotation has yet to be completed. This is essential to facilitate interrogation of the pig genome and investigation of less characterized genes. Efforts to develop a complete porcine proteome map as well as epigenome map are currently underway (Meurens et al., 2012; Schook et al., 2015a). These databases are necessary to understand disease pathogenesis (Meurens et al., 2012; Schook et al., 2015a). Moreover, the availability of both inbred and outbred breeds of minipigs extends the utility of these species as a viable large animal model. Continuing refinements and adaptation of technologies for genome editing, cell/tissue-specific gene targeting strategies, stem cells and somatic cell cloning will further facilitate the creation of specialized pig strains for biomedical research.

## Conflict of Interest Statement

The authors declare that the research was conducted in the absence of any commercial or financial relationships that could be construed as a potential conflict of interest.

## References

[B1] Abukawa H. Terai H. Hannouche D. Vacanti J. P. Kaban L. B. Troulis M. J. (2003). Formation of a mandibular condyle in vitro by tissue engineering. *J. Oral Maxillofac. Surg.* 61 94–100. 10.1053/joms.2003.50015 12524615

[B2] Aigner B. Renner S. Kessler B. Klymiuk N. Kurome M. Wunsch A. (2010). Transgenic pigs as models for translational biomedical research. *J. Mol. Med. (Berl.)* 88 653–664. 10.1007/s00109-010-0610-9 20339830

[B3] Amills M. Clop A. Ramírez O. Pérez-Enciso M. (2010). “Origin and genetic diversity of pig breeds,” in *Encyclopedia of Life Sciences (eLS)* (Chichester: John Wiley & Sons Ltd). 10.1002/9780470015902.a0022884

[B4] Amin Z. Woo R. Danford D. A. Froemming S. E. Reddy V. M. Lof J. (2006). Robotically assisted perventricular closure of perimembranous ventricular septal defects: preliminary results in Yucatan pigs. *J. Thorac. Cardiovasc. Surg.* 131 427–432. 10.1016/j.jtcvs.2005.10.034 16434274

[B5] Bjarkam C. R. Nielsen M. S. Glud A. N. Rosendal F. Mogensen P. Bender D. (2008). Neuromodulation in a minipig MPTP model of Parkinson disease. *Br. J. Neurosurg.* 22(Suppl. 1) S9–S12. 10.1080/02688690802448285 19085346

[B6] Bollen P. Ellegaard L. (1997). The Gottingen minipig in pharmacology and toxicology. *Pharmacol. Toxicol.* 80(Suppl. 2) 3–4. 10.1111/j.1600-0773.1997.tb01980.x 9249853

[B7] Bosse M. Madsen O. Megens H. J. Frantz L. A. Paudel Y. Crooijmans R. P. (2014a). Hybrid origin of European commercial pigs examined by an in-depth haplotype analysis on chromosome 1. *Front. Genet.* 5:442 10.3389/fgene.2014.00442 PMC4283659 25601878

[B8] Bosse M. Megens H.-J. Frantz L. A. F. Madsen O. Larson G. Paudel Y. (2014b). Genomic analysis reveals selection for Asian genes in European pigs following human-mediated introgression. *Nat. Commun.* 5:4392 10.1038/ncomms5392 PMC4225517 25025832

[B9] Brevini T. A. Antonini S. Cillo F. Crestan M. Gandolfi F. (2007a). Porcine embryonic stem cells: facts, challenges and hopes. *Theriogenology* 68(Suppl. 1) S206–S213. 10.1016/j.theriogenology.2007.05.043 17582486

[B10] Brevini T. A. Tosetti V. Crestan M. Antonini S. Gandolfi F. (2007b). Derivation and characterization of pluripotent cell lines from pig embryos of different origins. *Theriogenology* 67 54–63. 10.1016/j.theriogenology.2006.09.019 17055567

[B11] Buchanan D. S. Stalder K. (2011). “Breeds of pigs,” in *The Genetics of the Pig* eds Rothschild M. F. Ruvinsky A. (Cambridge, MA: CAB International).

[B12] Burchett K. M. Yan Y. Ouellette M. M. (2014). Telomerase inhibitor Imetelstat (GRN163L) limits the lifespan of human pancreatic cancer cells. *PLoS ONE* 9:e85155 10.1371/journal.pone.0085155 PMC3883701 24409321

[B13] Carlson D. F. Tan W. Lillico S. G. Stverakova D. Proudfoot C. Christian M. (2012). Efficient TALEN-mediated gene knockout in livestock. *Proc. Natl. Acad. Sci. U.S.A.* 109 17382–17387. 10.1073/pnas.1211446109 23027955 PMC3491456

[B14] Chia R. Achilli F. Festing M. F. Fisher E. M. (2005). The origins and uses of mouse outbred stocks. *Nat. Genet.* 37 1181–1186. 10.1038/ng1665 16254564

[B15] Christoffersen B. Golozoubova V. Pacini G. Svendsen O. Raun K. (2013). The young Gottingen minipig as a model of childhood and adolescent obesity: influence of diet and gender. *Obesity (Silver Spring)* 21 149–158. 10.1002/oby.20249 23505180

[B16] Cong L. Ran F. A. Cox D. Lin S. Barretto R. Habib N. (2013). Multiplex genome engineering using CRISPR/Cas systems. *Science* 339 819–823. 10.1126/science.1231143 23287718 PMC3795411

[B17] Cooper D. K. Gollackner B. Sachs D. H. (2002). Will the pig solve the transplantation backlog? *Annu. Rev. Med.* 53 133–147. 10.1146/annurev.med.53.082901.103900 11818467

[B18] Dalgaard L. (2014). Comparison of minipig, dog, monkey and human drug metabolism and disposition. *J. Pharmacol. Toxicol. Methods* 74 80–92. 10.1016/j.vascn.2014.12.005 25545337

[B19] Deponti D. Di Giancamillo A. Gervaso F. Domenicucci M. Domeneghini C. Sannino A. (2014). Collagen scaffold for cartilage tissue engineering: the benefit of fibrin glue and the proper culture time in an infant cartilage model. *Tissue Eng. Part A* 20 1113–1126. 10.1089/ten.TEA.2013.0171 24152291

[B20] Dexter J. D. Tumbleson M. E. Hutcheson D. P. Middleton C. C. (1976). Sinclair(S-1) miniature swine as a model for the study of human alcoholism. *Ann. N. Y. Acad. Sci.* 273 188–193. 10.1111/j.1749-6632.1976.tb52881.x 1072348

[B21] Dicks N. (2015). “Somatic cell nuclear transfer and the creation of transgenic large animal models,” in *Somatic Genome Manipulation: Advances, Methods and Applications* eds Donnelly D. J. Li X.-Q. Jensen T. G. (New York: Springer) 123–143.

[B22] Do M. Jang W. G. Hwang J. H. Jang H. Kim E. J. Jeong E. J. (2012). Inheritance of mitochondrial DNA in serially recloned pigs by somatic cell nuclear transfer (SCNT). *Biochem. Biophys. Res. Commun.* 424 765–770. 10.1016/j.bbrc.2012.07.031 22809505

[B23] Eggleston T. A. Roach W. P. Mitchell M. A. Smith K. Oler D. Johnson T. E. (2000). Comparison of two porcine (*Sus scrofa* domestica) skin models for in vivo near-infrared laser exposure. *Comp. Med.* 50 391–397. 11020157

[B24] England D. C. Panepinto L. M. (1986). “Conceptual and operational history of the development of miniature swine,” in *Swine in Biomedical Research* ed. Tumbleson M. E. (New York, NY: Plenum Press).

[B25] Esteban M. A. Xu J. Yang J. Peng M. Qin D. Li W. (2009). Generation of induced pluripotent stem cell lines from Tibetan miniature pig. *J. Biol. Chem.* 284 17634–17640. 10.1074/jbc.M109.008938 19376775 PMC2719402

[B26] Estrada J. L. Collins B. York A. Bischoff S. Sommer J. Tsai S. (2008). Successful cloning of the Yucatan minipig using commercial/occidental breeds as oocyte donors and embryo recipients. *Cloning Stem Cells* 10 287–296. 10.1089/clo.2008.0005 18373474 PMC2981378

[B27] Ezashi T. Telugu B. P. Alexenko A. P. Sachdev S. Sinha S. Roberts R. M. (2009). Derivation of induced pluripotent stem cells from pig somatic cells. *Proc. Natl. Acad. Sci. U.S.A.* 106 10993–10998. 10.1073/pnas.0905284106 19541600 PMC2698893

[B28] Ezashi T. Telugu B. P. Roberts R. M. (2012). Induced pluripotent stem cells from pigs and other ungulate species: an alternative to embryonic stem cells? *Reprod. Domest. Anim.* 47(Suppl. 4) 92–97. 10.1111/j.1439-0531.2012.02061.x 22827356

[B29] Feng W. Dai Y. Mou L. Cooper D. K. Shi D. Cai Z. (2015). The Potential of the Combination of CRISPR/Cas9 and Pluripotent Stem Cells to Provide Human Organs from Chimaeric Pigs. *Int. J. Mol. Sci.* 16 6545–6556. 10.3390/ijms16036545 25807262 PMC4394547

[B30] Festing M. F. (2014). Evidence should trump intuition by preferring inbred strains to outbred stocks in preclinical research. *ILAR J.* 55 399–404. 10.1093/ilar/ilu036 25541542

[B31] Flisikowska T. Merkl C. Landmann M. Eser S. Rezaei N. Cui X. (2012). A porcine model of familial adenomatous polyposis. *Gastroenterology* 143 e1171–e1177. 10.1053/j.gastro.2012.07.110 22864254

[B32] Giuffra E. Kijas J. M. Amarger V. Carlborg O. Jeon J. T. Andersson L. (2000). The origin of the domestic pig: independent domestication and subsequent introgression. *Genetics* 154 1785–1791. 10747069 10.1093/genetics/154.4.1785 PMC1461048

[B33] Gonzalez K. D. Noltner K. A. Buzin C. H. Gu D. Wen-Fong C. Y. Nguyen V. Q. (2009). Beyond Li Fraumeni Syndrome: clinical characteristics of families with p53 germline mutations. *J. Clin. Oncol.* 27 1250–1256. 10.1200/JCO.2008.16.6959 19204208

[B34] Gröger A. Kläring S. Merten H.-A. Holste J. Kaps C. Sittinger M. (2003). Tissue engineering of bone for mandibular augmentation in immunocompetent minipigs: preliminary study. *Scand. J. Plastic Reconstruct. Surg. Hand Surg.* 37 129–133. 10.1080/02844310310007728 12841611

[B35] Gun G. Kues W. A. (2014). Current progress of genetically engineered pig models for biomedical research. *Biores. Open. Access.* 3 255–264. 10.1089/biores.2014.0039 25469311 PMC4245835

[B36] Hai T. Teng F. Guo R. Li W. Zhou Q. (2014). One-step generation of knockout pigs by zygote injection of CRISPR/Cas system. *Cell Res.* 24 372–375. 10.1038/cr.2014.11 24481528 PMC3945887

[B37] Hauschild J. Petersen B. Santiago Y. Queisser A. L. Carnwath J. W. Lucas-Hahn A. (2011). Efficient generation of a biallelic knockout in pigs using zinc-finger nucleases. *Proc. Natl. Acad. Sci. U.S.A.* 108 12013–12017. 10.1073/pnas.1106422108 21730124 PMC3141985

[B38] Hedley P. L. Jorgensen P. Schlamowitz S. Moolman-Smook J. Kanters J. K. Corfield V. A. (2009). The genetic basis of Brugada syndrome: a mutation update. *Hum. Mutat.* 30 1256–1266. 10.1002/humu.21066 19606473

[B39] Huppertz N. Tolba R. Grosse J. (2015). Micturition in Gottingen minipigs: first reference in vivo data for urological research and review of literature. *Lab. Anim.* 10.1177/0023677215570993 [Epub ahead of print]. 25660835

[B40] Jeong Y. H. Park C. H. Jang G. H. Jeong Y. I. Hwang I. S. Jeong Y. W. (2013). Production of multiple transgenic Yucatan miniature pigs expressing human complement regulatory factors, human CD55. CD59, and H-transferase genes. *PLoS ONE* 8:e63241 10.1371/journal.pone.0063241 PMC3660325 23704897

[B41] Jiang J. Wang J. Wang H. Zhang Y. Kang H. Feng X. (2014). Global copy number analyses by next generation sequencing provide insight into pig genome variation. *BMC Genomics* 15:593 10.1186/1471-2164-15-593 PMC4111851 25023178

[B42] Johansen T. Hansen H. S. Richelsen B. Malmlof R. (2001). The obese Gottingen minipig as a model of the metabolic syndrome: dietary effects on obesity, insulin sensitivity, and growth hormone profile. *Comp. Med.* 51 150–155. 11922179

[B43] Jones G. F. (1998). “Genetic aspects of domestication, common breeds their origin,” in *The Genetics of the Pig* eds Rothschild M. F. Ruvinsky A. (New York: CAB International).

[B44] Klose R. Kemter E. Bedke T. Bittmann I. Keler B. Endres R. (2005). Expression of biologically active human TRAIL in transgenic pigs. *Transplantation* 80 222–230. 10.1097/01.tp.0000164817.59006.c2 16041267

[B45] Klymiuk N. Bocker W. Schonitzer V. Bahr A. Radic T. Frohlich T. (2012). First inducible transgene expression in porcine large animal models. *FASEB J.* 26 1086–1099. 10.1096/fj.11-185041 22138035

[B46] Köhn F. (2011). “History and development of miniature, micro- and minipigs,” in *The Minipig in Biomedical Research* eds Mcanulty P. A. Dayan A. D. Ganderup N. C. Hastings K. L. (Boca Raton, FL: CRC Press).

[B47] Koopmans S. J. Schuurman T. (2015). Considerations on pig models for appetite, metabolic syndrome and obese type 2 diabetes: from food intake to metabolic disease. *Eur. J. Pharmacol.* 759 231–239. 10.1016/j.ejphar.2015.03.044 25814261

[B48] Kornum B. R. Stott S. R. Mattsson B. Wisman L. Ettrup A. Hermening S. (2010). Adeno-associated viral vector serotypes 1 and 5 targeted to the neonatal rat and pig striatum induce widespread transgene expression in the forebrain. *Exp. Neurol.* 222 70–85. 10.1016/j.expneurol.2009.12.009 20025873

[B49] Kues W. A. Schwinzer R. Wirth D. Verhoeyen E. Lemme E. Herrmann D. (2006). Epigenetic silencing and tissue independent expression of a novel tetracycline inducible system in double-transgenic pigs. *FASEB J.* 20 1200–1202. 10.1096/fj.05-5415fje 16684801

[B50] Kurome M. Ishikawa T. Tomii R. Ueno S. Shimada A. Yazawa H. (2008). Production of transgenic and non-transgenic clones in miniature pigs by somatic cell nuclear transfer. *J. Reprod. Dev.* 54 156–163. 10.1262/jrd.20038 18296867

[B51] Kuzmuk K. N. Schook L. B. (2011). “Pig as a model for biomedical sciences,” in *The Genetics of the Pig* eds Rothschild M. F. Ruvinsky A. (Cambridge, MA: CAB International).

[B52] Kwon D. N. Lee K. Kang M. J. Choi Y. J. Park C. Whyte J. J. (2013). Production of biallelic CMP-Neu5Ac hydroxylase knock-out pigs. *Sci. Rep.* 3 1981 10.1038/srep01981 PMC4070623 23760311

[B53] Lai L. Kolber-Simonds D. Park K. W. Cheong H. T. Greenstein J. L. Im G. S. (2002). Production of alpha-1,3-galactosyltransferase knockout pigs by nuclear transfer cloning. *Science* 295 1089–1092. 10.1126/science.1068228 11778012

[B54] Larsen M. O. Wilken M. Gotfredsen C. F. Carr R. D. Svendsen O. Rolin B. (2002). Mild streptozotocin diabetes in the Gottingen minipig. A novel model of moderate insulin deficiency and diabetes. *Am. J. Physiol. Endocrinol. Metab.* 282 E1342–E1351. 10.1152/ajpendo.00564.2001 12006365

[B55] Larson G. Cucchi T. Dobney K. (2011). “Genetic aspects of pig domestication,” in *The Genetics of the Pig* eds Rothschild M. F. Ruvinsky A. (Cambridge, MA: CAB International).

[B56] Leal A. J. Tannuri A. C. Belon A. R. Guimaraes R. R. Coelho M. C. Goncalves Jde O. (2015). Effects of ischemic preconditioning in a pig model of large-for-size liver transplantation. *Clinics (Sao Paulo)* 70 126–135. 10.6061/clinics/2015(02)10 25789522 PMC4351307

[B57] Lee K. Kwon D. N. Ezashi T. Choi Y. J. Park C. Ericsson A. C. (2014). Engraftment of human iPS cells and allogeneic porcine cells into pigs with inactivated RAG2 and accompanying severe combined immunodeficiency. *Proc. Natl. Acad. Sci. U.S.A.* 111 7260–7265. 10.1073/pnas.1406376111 24799706 PMC4034252

[B58] Leigh H. Forbes P. D. Lawson C. Kim D. Y. White D. Brown L. D. (2012). Miniature swine model of phototoxicity testing. *Photodermatol. Photoimmunol. Photomed.* 28 34–41. 10.1111/j.1600-0781.2011.00633.x 22212001

[B59] Leuchs S. Saalfrank A. Merkl C. Flisikowska T. Edlinger M. Durkovic M. (2012). Inactivation and inducible oncogenic mutation of p53 in gene targeted pigs. *PLoS ONE* 7:e43323 10.1371/journal.pone.0043323 PMC3465291 23071491

[B60] Li L. Pang D. Wang T. Li Z. Chen L. Zhang M. (2009). Production of a reporter transgenic pig for monitoring Cre recombinase activity. *Biochem. Biophys. Res. Commun.* 382 232–235. 10.1016/j.bbrc.2009.02.146 19268654

[B61] Li X. Zhou X. Guan Y. Wang Y. X. Scutt D. Gong Q. Y. (2006). N-nitrosodiethylamine-induced pig liver hepatocellular carcinoma model: radiological and histopathological studies. *Cardiovasc. Intervent. Radiol.* 29 420–428. 10.1007/s00270-005-0099-8 16502159

[B62] Luo W. Li Z. Huang Y. Han Y. Yao C. Duan X. (2014). Generation of AQP2-Cre transgenic mini-pigs specifically expressing Cre recombinase in kidney collecting duct cells. *Transg. Res.* 23 365–375. 10.1007/s11248-013-9774-8 24307331

[B63] Luo Y. Bolund L. Sorensen C. B. (2012). Pig gene knockout by rAAV-mediated homologous recombination: comparison of BRCA1 gene knockout efficiency in Yucatan and Gottingen fibroblasts with slightly different target sequences. *Transg. Res.* 21 671–676. 10.1007/s11248-011-9563-1 22020980

[B64] Luo Y. Li J. Liu Y. Lin L. Du Y. Li S. (2011). High efficiency of BRCA1 knockout using rAAV-mediated gene targeting: developing a pig model for breast cancer. *Transg. Res.* 20 975–988. 10.1007/s11248-010-9472-8 21181439

[B65] Matsunari H. Nagashima H. Watanabe M. Umeyama K. Nakano K. Nagaya M. (2013). Blastocyst complementation generates exogenic pancreas in vivo in apancreatic cloned pigs. *Proc. Natl. Acad. Sci. U.S.A.* 110 4557–4562. 10.1073/pnas.1222902110 23431169 PMC3607052

[B66] McGlone J. Pond W. (2003). *Pig Production: Biological Principles and Applications*. Florence: Thomson - Delmar Learning.

[B67] Meurens F. Summerfield A. Nauwynck H. Saif L. Gerdts V. (2012). The pig: a model for human infectious diseases. *Trends Microbiol.* 20 50–57. 10.1016/j.tim.2011.11.002 22153753 PMC7173122

[B68] Millikan L. E. Boylon J. L. Hook R. R. Manning P. J. (1974). Melanoma in Sinclair swine: a new animal model. *J. Invest. Dermatol.* 62 20–30. 10.1111/1523-1747.ep12676714 4809019

[B69] Mitra A. Leyes A. Manser K. Roadcap B. Mestre C. Tatosian D. (2015). Use of minipig skin biopsy model as an innovative tool to design topical formulation to achieve desired pharmacokinetics in humans. *J. Pharm. Sci.* 104 1701–1708. 10.1002/jps.24383 25691117

[B70] Miyagawa S. Murakami H. Takahagi Y. Nakai R. Yamada M. Murase A. (2001). Remodeling of the major pig xenoantigen by N-acetylglucosaminyltransferase III in transgenic pig. *J. Biol. Chem.* 276 39310–39319. 10.1074/jbc.M104359200 11486004

[B71] Murakami H. Nagashima H. Takahagi Y. Miyagawa S. Fujimura T. Toyomura K. (2002). Transgenic pigs expressing human decay-accelerating factor regulated by porcine MCP gene promoter. *Mol. Reprod. Dev.* 61 302–311. 10.1002/mrd.10043 11835575

[B72] Neeb Z. P. Edwards J. M. Alloosh M. Long X. Mokelke E. A. Sturek M. (2010). Metabolic syndrome and coronary artery disease in Ossabaw compared with Yucatan swine. *Comp. Med.* 60 300–315. 20819380 PMC2930329

[B73] Newman S. J. Rohrbach B. (2012). Pot-bellied pig neoplasia: a retrospective case series (2004-2011). *J. Vet. Diagn. Invest.* 24 1008–1013. 10.1177/1040638712452725 22826040

[B74] Nielsen V. H. Bendixen C. Arnbjerg J. Sorensen C. M. Jensen H. E. Shukri N. M. (2000). Abnormal growth plate function in pigs carrying a dominant mutation in type X collagen. *Mamm. Genome* 11 1087–1092. 10.1007/s003350010212 11130976

[B75] Onishi A. Iwamoto M. Akita T. Mikawa S. Takeda K. Awata T. (2000). Pig cloning by microinjection of fetal fibroblast nuclei. *Science* 289 1188–1190. 10.1126/science.289.5482.1188 10947985

[B76] Oxenhandler R. W. Adelstein E. H. Haigh J. P. Hook R. R. Jr. Clark W. H. Jr. (1979). Malignant melanoma in the Sinclair miniature swine: an autopsy study of 60 cases. *Am. J. Pathol.* 96 707–720. 474716 PMC2042391

[B77] Panepinto L. M. (1996). “Miniature swine breeds used worldwide in research,” in *Advances in Swine in Biomedical Research* eds Tumbleson M. E. Schook L. B. (New York, NY: Plenum Press).

[B78] Park D. S. Cerrone M. Morley G. Vasquez C. Fowler S. Liu N. (2015). Genetically engineered SCN5A mutant pig hearts exhibit conduction defects and arrhythmias. *J. Clin. Invest.* 125 403–412. 10.1172/JCI76919 25500882 PMC4382241

[B79] Patel A. S. Saeed M. Yee E. J. Yang J. Lam G. J. Losey A. D. (2014). Development and validation of endovascular chemotherapy filter device for removing high-dose doxorubicin: preclinical study. *J. Med. Device.* 8 0410081–0410088. 10.1115/1.4027444 25653735 PMC4298098

[B80] Pathak S. Multani A. S. McConkey D. J. Imam A. S. Amoss M. S. Jr. (2000). Spontaneous regression of cutaneous melanoma in sinclair swine is associated with defective telomerase activity and extensive telomere erosion. *Int. J. Oncol.* 17 1219–1224. 11078808 10.3892/ijo.17.6.1219

[B81] Petersen B. Ramackers W. Lucas-Hahn A. Lemme E. Hassel P. Queisser A. L. (2011). Transgenic expression of human heme oxygenase-1 in pigs confers resistance against xenograft rejection during ex vivo perfusion of porcine kidneys. *Xenotransplantation* 18 355–368. 10.1111/j.1399-3089.2011.00674.x 22168142

[B82] Phelps C. J. Ball S. F. Vaught T. D. Vance A. M. Mendicino M. Monahan J. A. (2009). Production and characterization of transgenic pigs expressing porcine CTLA4-Ig. *Xenotransplantation* 16 477–485. 10.1111/j.1399-3089.2009.00533.x 20042047

[B83] Phelps C. J. Koike C. Vaught T. D. Boone J. Wells K. D. Chen S. H. (2003). Production of alpha 1,3-galactosyltransferase-deficient pigs. *Science* 299 411–414. 10.1126/science.1078942 12493821 PMC3154759

[B84] Phillips R. W. Panepinto L. M. Spangler R. Westmoreland N. (1982). Yucatan miniature swine as a model for the study of human diabetes mellitus. *Diabetes Metab. Res. Rev.* 31 30–36. 10.2337/diab.31.1.s30 6761193

[B85] Phillips R. W. Panepinto L. M. Will D. H. Case G. L. (1980). The effects of alloxan diabetes on Yucatan miniature swine and their progeny. *Metabolism* 29 40–45. 10.1016/0026-0495(80)90096-7 7188711

[B86] Polejaeva I. A. Chen S.-H. Vaught T. D. Page R. L. Mullins J. Ball S. (2000). Cloned pigs produced by nuclear transfer from adult somatic cells. *Nature* 407 86–90. 10.1038/35024082 10993078

[B87] Porter V. (1993). *Pigs: A Handbook to the Breeds of the World*. Ithaca, NY: Cornell University Press.

[B88] Prather R. S. Lorson M. Ross J. W. Whyte J. J. Walters E. (2013). Genetically engineered pig models for human diseases. *Annu. Rev. Anim. Biosci.* 1 203–219. 25387017 10.1146/annurev-animal-031412-103715 PMC4460601

[B89] Purohit D. M. Swindle M. M. Smith C. D. Othersen H. B. Jr. Kazanovicz J. M. (1993). Hanford miniature swine model for extracorporeal membrane oxygenation. *J. Invest. Surg.* 6 503–508. 10.3109/08941939309141640 8123611

[B90] Reger S. I. Hyodo A. Negami S. Kambic H. E. Sahgal V. (1999). Experimental wound healing with electrical stimulation. *Artif. Organs.* 23 460–462. 10.1046/j.1525-1594.1999.06365.x 10378943

[B91] Rogers C. S. Stoltz D. A. Meyerholz D. K. Ostedgaard L. S. Rokhlina T. Taft P. J. (2008). Disruption of the CFTR gene produces a model of cystic fibrosis in newborn pigs. *Science* 321 1837–1841. 10.1126/science.1163600 18818360 PMC2570747

[B92] Ropka-Molik K. Zukowski K. Eckert R. Gurgul A. Piorkowska K. Oczkowicz M. (2014). Comprehensive analysis of the whole transcriptomes from two different pig breeds using RNA-Seq method. *Anim. Genet.* 45 674–684. 10.1111/age.12184 24961663

[B93] Rosati R. Horan G. S. Pinero G. J. Garofalo S. Keene D. R. Horton W. A. (1994). Normal long bone growth and development in type X collagen-null mice. *Nat. Genet.* 8 129–135. 10.1038/ng1094-129 7842010

[B94] Safranski T. J. (2008). Genetic selection of boars. *Theriogenology* 70 1310–1316. 10.1016/j.theriogenology.2008.06.020 18672281

[B95] Sahana G. Kadlecova V. Hornshoj H. Nielsen B. Christensen O. F. (2013). A genome-wide association scan in pig identifies novel regions associated with feed efficiency trait. *J. Anim. Sci.* 91 1041–1050. 10.2527/jas.2012-5643 23296815

[B96] Sanchez M. P. Tribout T. Iannuccelli N. Bouffaud M. Servin B. Tenghe A. (2014). A genome-wide association study of production traits in a commercial population of Large White pigs: evidence of haplotypes affecting meat quality. *Genet. Sel. Evol.* 46 12 10.1186/1297-9686-46-12 PMC3975960 24528607

[B97] Sangild P. T. Ney D. M. Sigalet D. L. Vegge A. Burrin D. (2014). Animal models of gastrointestinal and liver diseases. Animal models of infant short bowel syndrome: translational relevance and challenges. *Am. J. Physiol. Gastrointest. Liver Physiol.* 307 G1147–G1168. 10.1152/ajpgi.00088.2014 25342047 PMC4269678

[B98] Schook L. B. Collares T. V. Darfour-Oduro K. A. De A. K. Rund L. A. Schachtschneider K. M. (2015a). Unraveling the Swine genome: implications for human health. *Annu. Rev. Anim. Biosci.* 3 219–244. 10.1146/annurev-animal-022114-110815 25689318

[B99] Schook L. B. Collares T. V. Hu W. Liang Y. Rodrigues F. M. Rund L. A. (2015b). A genetic porcine model of cancer. *PLoS ONE* 10:e0128864 10.1371/journal.pone.0128864 PMC4488487 26132737

[B100] Schuleri K. H. Boyle A. J. Centola M. Amado L. C. Evers R. Zimmet J. M. (2008). The adult Gottingen minipig as a model for chronic heart failure after myocardial infarction: focus on cardiovascular imaging and regenerative therapies. *Comp. Med.* 58 568–579. 19149414 PMC2710749

[B101] Seok J. Warren H. S. Cuenca A. G. Mindrinos M. N. Baker H. V. Xu W. (2013). Genomic responses in mouse models poorly mimic human inflammatory diseases. *Proc. Natl. Acad. Sci. U.S.A.* 110 3507–3512. 10.1073/pnas.1222878110 23401516 PMC3587220

[B102] Sieren J. C. Meyerholz D. K. Wang X. J. Davis B. T. Newell J. D. Jr. Hammond E. (2014). Development and translational imaging of a TP53 porcine tumorigenesis model. *J. Clin. Invest.* 124 4052–4066. 10.1172/JCI75447 25105366 PMC4151205

[B103] Snouwaert J. N. Brigman K. K. Latour A. M. Malouf N. N. Boucher R. C. Smithies O. (1992). An animal model for cystic fibrosis made by gene targeting. *Science* 257 1083–1088. 10.1126/science.257.5073.1083 1380723

[B104] Spurlock M. E. Gabler N. K. (2008). The development of porcine models of obesity and the metabolic syndrome. *J. Nutr.* 138 397–402. 18203910 10.1093/jn/138.2.397

[B105] Su L. K. Kinzler K. W. Vogelstein B. Preisinger A. C. Moser A. R. Luongo C. (1992). Multiple intestinal neoplasia caused by a mutation in the murine homolog of the APC gene. *Science* 256 668–670. 10.1126/science.256.5060.1114-c 1350108

[B106] Sullivan T. P. Eaglstein W. H. Davis S. C. Mertz P. (2001). The pig as a model for human wound healing. *Wound Repair. Regen.* 9 66–76. 10.1046/j.1524-475x.2001.00066.x 11350644

[B107] Swindle M. M. Makin A. Herron A. J. Clubb F. J. Jr. Frazier K. S. (2012). Swine as models in biomedical research and toxicology testing. *Vet. Pathol.* 49 344–356. 10.1177/0300985811402846 21441112

[B108] Swindle M. M. Thompson R. P. Carabello B. A. Smith A. C. Hepburn B. J. Bodison D. R. (1990). Heritable ventricular septal defect in Yucatan miniature swine. *Lab. Anim. Sci.* 40 155–161. 2157094

[B109] Takahagi Y. Fujimura T. Miyagawa S. Nagashima H. Shigehisa T. Shirakura R. (2005). Production of alpha 1,3-galactosyltransferase gene knockout pigs expressing both human decay-accelerating factor and N-acetylglucosaminyltransferase III. *Mol. Reprod. Dev.* 71 331–338. 10.1002/mrd.20305 15806557

[B110] Tank J. C. Weiner D. S. Jacquet R. Childs D. Ritzman T. F. Horne W. I. (2013). The effects of hypothyroidism on the proximal femoral physis in miniature swine. *J. Orthop. Res.* 31 1986–1991. 10.1002/jor.22467 24038610

[B111] Tart J. K. Johnson R. K. Bundy J. W. Ferdinand N. N. Mcknite A. M. Wood J. R. (2013). Genome-wide prediction of age at puberty and reproductive longevity in sows. *Anim. Genet.* 44 387–397. 10.1111/age.12028 23437861

[B112] Van Dyke T. E. Hasturk H. Kantarci A. Freire M. O. Nguyen D. Dalli J. (2015). Proresolving nanomedicines activate bone regeneration in periodontitis. *J. Dent. Res.* 94 148–156. 10.1177/0022034514557331 25389003 PMC4270812

[B113] van Mierlo G. J. Cnubben N. H. Kuper C. F. Wolthoorn J. Van Meeteren-Kreikamp A. P. Nagtegaal M. M. (2013). The Gottingen minipig(R) as an alternative non-rodent species for immunogenicity testing: a demonstrator study using the IL-1 receptor antagonist anakinra. *J. Immunotoxicol.* 10 96–105. 10.3109/1547691X.2012.735274 23134195

[B114] Wakai T. Sugimura S. Yamanaka K. Kawahara M. Sasada H. Tanaka H. (2008). Production of viable cloned miniature pig embryos using oocytes derived from domestic pig ovaries. *Cloning Stem Cells* 10 249–262. 10.1089/clo.2007.0045 18352818

[B115] Warman M. L. Abbott M. Apte S. S. Hefferon T. Mcintosh I. Cohn D. H. (1993). A type X collagen mutation causes Schmid metaphyseal chondrodysplasia. *Nat. Genet.* 5 79–82. 10.1038/ng0993-79 8220429

[B116] West F. D. Terlouw S. L. Kwon D. J. Mumaw J. L. Dhara S. K. Hasneen K. (2010). Porcine induced pluripotent stem cells produce chimeric offspring. *Stem Cells Dev.* 19 1211–1220. 10.1089/scd.2009.0458 20380514

[B117] West F. D. Uhl E. W. Liu Y. Stowe H. Lu Y. Yu P. (2011). Brief report: chimeric pigs produced from induced pluripotent stem cells demonstrate germline transmission and no evidence of tumor formation in young pigs. *Stem Cells* 29 1640–1643. 10.1002/stem.713 22039609

[B118] Whitworth K. M. Lee K. Benne J. A. Beaton B. P. Spate L. D. Murphy S. L. (2014). Use of the CRISPR/Cas9 system to produce genetically engineered pigs from in vitro-derived oocytes and embryos. *Biol. Reprod.* 91 78 10.1095/biolreprod.114.121723 PMC4435063 25100712

[B119] Whyte J. J. Prather R. S. (2011). Genetic modifications of pigs for medicine and agriculture. *Mol. Reprod. Dev.* 78 879–891. 10.1002/mrd.21333 21671302 PMC3522184

[B120] Xi S. Yin W. Wang Z. Kusunoki M. Lian X. Koike T. (2004). A minipig model of high-fat/high-sucrose diet-induced diabetes and atherosclerosis. *Int. J. Exp. Pathol.* 85 223–231. 10.1111/j.0959-9673.2004.00394.x 15312127 PMC2517483

[B121] Zhao J. Ross J. W. Hao Y. Spate L. D. Walters E. M. Samuel M. S. (2009). Significant improvement in cloning efficiency of an inbred miniature pig by histone deacetylase inhibitor treatment after somatic cell nuclear transfer. *Biol. Reprod.* 81 525–530. 10.1095/biolreprod.109.077016 19386991 PMC2731980

